# A case-control study of the association between the *EGFR* gene and glioma risk in a Chinese Han population

**DOI:** 10.18632/oncotarget.16946

**Published:** 2017-04-07

**Authors:** Mengdan Yan, Jingjie Li, Na He, Xugang Shi, Shuli Du, Bin Li, Tianbo Jin

**Affiliations:** ^1^ Key Laboratory of Resource Biology and Biotechnology in Western China, Northwest University, Ministry of Education, Xi’an, China; ^2^ Xi’an Tiangen Precision Medical Research Institute, Xi’an, China; ^3^ Department of Neurosurgery, Xi’an First Hospital, Xi’an, China

**Keywords:** glioma, EGFR gene, tag single nucleotide polymorphism, case–control study

## Abstract

The Epidermal Growth Factor Receptor gene has been reported to be involved in the progression of gliomas which is one of the deadliest primary brain tumors in humans. To determine potential association between *EGFR* and glioma risk, we performed a case-control study with 394 glioma patients and 298 cancer-free controls in which captured a total of 8 tag single nucleotide polymorphisms of *EGFR* gene from Xi’an, China. SPSS 19.0 statistical packages, χ^2^ test, genetic model analysis and SHEsis software platform were analyzed s the variants in *EGFR* gene associations with glioma risk. For five different inheritance models analyzed, the following genotypes were associated with increased glioma risk. In the codominant model, genotype CC (rs730437, OR = 1.93, *p* = 0.024; rs1468727, OR = 2.02, *p* = 0.007); In the dominant model, genotype CA and CC (rs730437, OR = 1.45, *p* = 0.026), genotype GA and AA (rs845552, OR = 1.40, *p* = 0.044); In the recessive model, genotype CC (rs730437, OR = 1.64, *p* = 0.026; rs1468727, OR = 1.87, *p* = 0.002); In the additive model, genotype CC (rs730437, OR = 1.32, *p* = 0.006; rs1468727, OR = 1.39, *p* = 0.005), genotype GG (rs11506105, OR = 1.32, *p* = 0.02) and genotype AA (rs845552, OR = 1.27, *p* = 0.04). Our study indicated that 8 mutants located in *EGFR* gene were risk-conferring factors, larger and different populations with *EGFR* polymorphisms are required to verify these associations.

## INTRODUCTION

Glioma is the most common primary central nervous system (CNS) tumors that originate from glia, accounting for approximately 80% of all primary malignant brain tumors, with nearly 20,000 people affected annually in the United States [1]. The survival time and prognosis of patients diagnosed with gliomas in adults is very poor. According to the WHO classification (2007), gliomas are classified into four grades (I to IV) based on microscopic appearance [2]. Alternatively, they are divided into astrocytic tumors, oligodendrogliomas, and oligoastrocytomas. Despite high-dosage ionizing irradiation (IR) devoted to the risk factor for gliomas, the etiology of sporadic gliomas remains unexplained [3]. In addition, only a small proportion of individuals who are exposed to IR environments will develop gliomas, suggesting hereditary factors contribute to susceptibility to glioma.

The Epidermal Growth Factor Receptor (*EGFR*) is a transmembrane tyrosine kinase in 7p11.2, It is one of the critical oncogenes for several cancers and variably expressed from embryogenesis to adulthood in the normal brain development. For low-grade glioma [4] and anaplastic astrocytoma [5], The overexpression of EGFR may be a prominent event in the progression of gliomas with a poor prognosis. Current evidence suggests that *EGFR* amplification is a hallmark of glioblastoma (GBM). As the most frequent and the most malignant glioma in the adult population [6–8], accounting for approximately 30% - 50% of high-grade glioma and containing a genetic mutation of *EGFR* (*EGFR*vIII).

Although several studies showed that *EGFR* gene polymorphisms were associated with glioma risk, those currently available results are inconclusive [9–11]. The aim of this study was to investigate and validate the potential relationship between tSNPs in *EGFR* gene and glioma susceptibility in the Han Chinese population using a case-control study.

## RESULTS

### Study participants

The details of selected characteristics of all subjects were presented in Table 1, including gender, age, and histologic type. We enrolled a total of 692 participants, including 394 patients (211 males, 183 females) and 298 controls (119 males, 179 females) in this study, the mean age of the subjects was 42.64 ± 16.89 years for cases and 49.91 ± 7.58 years for controls. The distributions of gender and age were statistically significant differences between the case and control groups (p < 0.05). Of the cancers, astrocytoma (175) and glioblastoma (116) are the more common histologies, and ependymoma (19), oligodendroglioma (10), oligodendrocytes astrocytoma (35) and others (39) are the less common histologies.

**Table 1:** General characteristics of glioma subjects and healthy controls

		Case (n = 394)		Control (n = 298)		*p* value from χ^2^
		Count	Percentage (%)	Count	Percentage (%)	
Gender						< 0.05
	male	211	53.60	119	39.90	
	female	183	46.40	179	60.10	
Age						< 0.05
	Mean age	42.64 ± 16.89		49.91 ± 7.58		
Histologic type						
	Astrocytoma	175	44.42			
	Ependymoma	19	4.82			
	Glioblastoma	116	29.44			
	Oligodendroglioma	10	2.54			
	Oligodendrocytes astrocytoma	35	8.88			
	others	39	9.90			

### Hardy–Weinberg equilibrium and alleles of eight tSNPs

A total of 8 tag single nucleotide polymorphisms (tSNPs) were successfully genotyped in all participants, and all of the tested tSNPs were in accordance with Hardy–Weinberg equilibrium (HWE) in control subjects (*p* > 0.05) .We assumed that the minor allele of each tSNP was a risk factor compared to the wild-type allele. MAF in cases and controls were showed in Table 2. Using the χ^2^ test, three tSNPs were found to be associated with glioma and risk at a 5% level (rs11506105, OR = 1.26, 95% CI = 1.01-1.57, *p* = 0.042; rs1468727, OR = 1.31, 95% CI = 1.05-1.62, *p* = 0.016; rs730437, OR = 1.33, 95% CI = 1.07-1.66, *p* = 0.01). Given the number of glioma subjects, we only performed the stratified analysis with more common histologies (astrocytoma and glioblastoma), and also detected that three tSNPs were associated with an increased risk of astrocytoma (rs11506105, OR = 1.32, 95% CI = 1.00-1.73, *p* = 0.046; rs1468727, OR = 1.37, 95% CI = 1.05-1.79, *p* = 0.02; rs730437, OR = 1.42, 95% CI = 1.09-1.86, *p* = 0.01). However, there was no association of all tSNPs with risk of glioblastoma (Table 3).

**Table 2:** Basic information about *EGFR* candidate tSNPs in this study

SNP ID	Location	Position	Gene	Role	Glioma frequency (MAF)		Glioblastoma frequency (MAF)		Astrocytoma frequency (MAF)		HWE *p*-value
					Case	Control	Case	Control	Case	Control	
rs11506105	7p11.2	55220177	EGFR	Intron (boundary)	0.41	0.356	0.414	0.586	0.171	0.829	0.898
rs12718945	7p11.2	55192963	EGFR	Intron	0.354	0.343	0.341	0.659	0.109	0.891	0.898
rs1468727	7p11.2	55230105	EGFR	Intron	0.496	0.43	0.478	0.522	0.169	0.831	0.278
rs17172432	7p11.2	55141317	EGFR	Intron	0.096	0.108	0.099	0.901	0.100	0.900	0.760
rs3752651	7p11.2	55229543	EGFR	Intron	0.083	0.074	0.073	0.927	0.355	0.645	1.000
rs4947492	7p11.2	55187992	EGFR	Intron	0.359	0.347	0.345	0.655	0.422	0.578	0.899
rs730437	7p11.2	55215018	EGFR	Intron	0.433	0.364	0.431	0.569	0.509	0.491	1.000
rs845552	7p11.2	55245507	EGFR	Intron	0.411	0.361	0.409	0.591	0.417	0.583	0.370

Abbreviations: MAF: minor allele frequency

**Table 3:** Association between tSNPs in EGFR gene and risk of different histological types of gliomas in allelic model analysis

SNP ID	Base change	Glioma			Astrocytoma				Glioblastoma		
		ORs (95%CI)^c^	*p*-value	*p* value adj. *	ORs (95%CI)	*p*-value	*p* value adj. *	ORs (95%CI)	*p*-value	*p* value adj. *
rs11506105	A/G	1.26(1.01-1.57)	0.042*	0.336	1.32(1.00-1.73)	0.046*	0.368	1.28(0.93-1.74)	0.125	1
rs12718945	G/T	1.05(0.84-1.32)	0.663	1	1.06(0.80-1.40)	0.696	1	0.99(0.72-1.36)	0.948	1
rs1468727	T/C	1.31(1.05-1.62)	0.016*	0.128	1.37(1.05-1.79)	0.020*	0.160	1.21(0.89-1.65)	0.217	1
rs17172432	T/C	0.88(0.62-1.25)	0.478	1	0.92(0.59-1.42)	0.695	1	0.91(0.55-1.50)	0.706	1
rs3752651	T/C	1.13(0.76-1.68)	0.556	1	1.14(0.70-1.85)	0.608	1	0.99(0.55-1.77)	0.969	1
rs4947492	A/G	1.06(0.84-1.32)	0.635	1	1.06(0.80-1.40)	0.682	1	0.99(0.72-1.36)	0.957	1
rs730437	A/C	1.33(1.07-1.66)	0.010*	0.080	1.42(1.09-1.86)	0.010*	0.080	1.32(0.97-1.80)	0.075	0.600
rs845552	G/A	1.24(0.99-1.54)	0.062	1	1.27(0.97-1.67)	0.086	0.688	1.23(0.90-1.68)	0.195	1

Abbreviations: OR: odds ratio; 95%CI: 95% confidence interval.

* p ≤ 0.05 indicates statistical significance.

*p value was adjusted by Bonferroni corrections.

### Association between EGFR and glioma risk

We further analyzed the association between *EGFR* tSNPs and glioma risk under five gene models (codominant, dominant, recessive, overdominant and additive). Table 4 showed that the genotypes of tSNPs were associated with increased glioma risk: in the codominant model, genotype CC (rs730437, OR = 1.93, 95%CI: 1.19-3.13, *p* = 0.024; rs1468727, OR = 2.02, 95%CI: 1.26-3.24, p = 0.007); In the dominant model, genotype CA and CC (rs730437, OR = 1.45, 95%CI: 1.05-2.02, *p* = 0.026), genotype GA and AA (rs845552, OR = 1.40, 95%CI: 1.01-1.94, *p* = 0.044); In the recessive model, genotype CC (rs730437, OR = 1.64, 95%CI: 1.06-2.56, *p* = 0.026; rs1468727, OR = 1.87, 95%CI: 1.25-2.81, *p* = 0.002); In the additive model, genotype CC (rs730437, OR = 1.37, 95%CI: 1.09-1.73, *p* = 0.006; rs1468727, OR = 1.39, 95%CI: 1.10-1.75, *p* = 0.005), genotype GG (rs11506105, OR = 1.32, 95%CI: 1.04-1.67, *p* = 0.02) and genotype AA (rs845552, OR = 1.27, 95%CI: 1.01-1.59, *p* = 0.04). In addition, we observed the association between the *EGFR* and increased astrocytoma risk (Table 5), in the codominant model, genotype CC (rs730437, OR = 2.27, 95%CI: 1.26-4.10, *p* = 0.024; rs1468727, OR = 2.09, 95%CI: 1.17-3.72, *p* = 0.022); In the recessive model, genotype CC (rs730437, OR = 2.00, 95%CI: 1.17-3.41, *p* = 0.011; rs1468727, OR = 1.99, 95%CI: 1.22-3.24, *p* = 0.0062) and genotype GG (rs11506105, OR = 1.86, 95%CI: 1.07-3.23, *p* = 0.029). In the additive model, genotype CC (rs730437, OR = 1.46, 95%CI: 1.10-1.95, *p* = 0.0095; rs1468727, OR = 1.42, 95%CI: 1.06-1.90, *p* = 0.018), genotype GG (rs11506105, OR = 1.34, 95%CI: 1.01-1.80, *p* = 0.045).

**Table 4:** Relationship between *EGFR* tSNPs and glioma cancer risk under multiple models of inheritance

SNP ID	Model	Genotype	control	case	crude analysis		adjusted by age and gender	
					OR (95% CI)	*p* -value	OR (95% CI)	*p* -value
rs730437	Codominant	A/A	120 (40.3%)	126 (32%)	1	0.035	1	0.024*
		C/A	139 (46.6%)	195 (49.5%)	1.34 (0.96-1.86)		1.33 (0.94-1.87)	
		C/C	39 (13.1%)	73 (18.5%)	1.78 (1.12-2.83)		1.93 (1.19-3.13)	
	Dominant	A/A	120 (40.3%)	126 (32%)	1	0.024	1	0.026*
		C/A-C/C	178 (59.7%)	268 (68%)	1.43 (1.05-1.96)		1.45 (1.05-2.02)	
	Recessive	A/A-C/A	259 (86.9%)	321 (81.5%)	1	0.052	1	0.026*
		C/C	39 (13.1%)	73 (18.5%)	1.51 (0.99-2.30)		1.64 (1.06-2.56)	
	Overdominant	A/A-C/C	159 (53.4%)	199 (50.5%)	1	0.460	1	0.600
		C/A	139 (46.6%)	195 (49.5%)	1.12 (0.83-1.52)		1.09 (0.79-1.49)	
	Log-additive	---	---	---	1.34 (1.07-1.66)	0.010	1.37 (1.09-1.73)	0.006*
rs11506105	Codominant	A/A	120 (41.1%)	134 (34.4%)	1	0.120	1	0.064
		A/G	136 (46.6%)	192 (49.2%)	1.26 (0.91-1.76)		1.28 (0.91-1.81)	
		G/G	36 (12.3%)	64 (16.4%)	1.59 (0.99-2.56)		1.78 (1.08-2.93)	
	Dominant	A/A	120 (41.1%)	134 (34.4%)	1	0.072	1	0.053
		A/G-G/G	172 (58.9%)	256 (65.6%)	1.33 (0.97-1.82)		1.38 (1.00-1.92)	
	Recessive	A/A-A/G	256 (87.7%)	326 (83.6%)	1	0.130	1	0.061
		G/G	36 (12.3%)	64 (16.4%)	1.40 (0.90-2.17)		1.55 (0.97-2.45)	
	Overdominant	A/A-G/G	156 (53.4%)	198 (50.8%)	1	0.490	1	0.570
		A/G	136 (46.6%)	192 (49.2%)	1.11 (0.82-1.51)		1.10 (0.80-1.51)	
	Log-additive	---	---	---	1.26 (1.01-1.58)	0.041	1.32 (1.04-1.67)	0.020*
rs1468727	Codominant	T/T	88 (30.8%)	102 (26.1%)	1	0.024	1	0.007*
		T/C	150 (52.5%)	190 (48.6%)	1.09 (0.77-1.56)		1.13 (0.77-1.64)	
		C/C	48 (16.8%)	99 (25.3%)	1.78 (1.14-2.78)		2.02 (1.26-3.24)	
	Dominant	T/T	88 (30.8%)	102 (26.1%)	1	0.180	1	0.110
		T/C-C/C	198 (69.2%)	289 (73.9%)	1.26 (0.90-1.77)		1.33 (0.93-1.90)	
	Recessive	T/T-T/C	238 (83.2%)	292 (74.7%)	1	0.007	1	0.002*
		C/C	48 (16.8%)	99 (25.3%)	1.68 (1.14-2.47)		1.87 (1.25-2.81)	
	Overdominant	T/T-C/C	136 (47.5%)	201 (51.4%)	1	0.320	1	0.280
		T/C	150 (52.5%)	190 (48.6%)	0.86 (0.63-1.16)		0.84 (0.61-1.15)	
	Log-additive	---	---	---	1.31 (1.05-1.63)	0.015	1.39 (1.10-1.75)	0.005*
rs845552	Codominant	G/G	121 (42.2%)	140 (35.7%)	1	0.180	1	0.110
		G/A	125 (43.5%)	182 (46.4%)	1.26 (0.90-1.76)		1.35 (0.95-1.92)	
		A/A	41 (14.3%)	70 (17.9%)	1.48 (0.94-2.33)		1.55 (0.96-2.51)	
	Dominant	G/G	121 (42.2%)	140 (35.7%)	1	0.088	1	0.044*
		G/A-A/A	166 (57.8%)	252 (64.3%)	1.31 (0.96-1.79)		1.40 (1.01-1.94)	
	Recessive	G/G-G/A	246 (85.7%)	322 (82.1%)	1	0.210	1	0.210
		A/A	41 (14.3%)	70 (17.9%)	1.30 (0.86-1.98)		1.32 (0.85-2.05)	
	Overdominant	G/G-A/A	162 (56.5%)	210 (53.6%)	1	0.460	1	0.300
		G/A	125 (43.5%)	182 (46.4%)	1.12 (0.83-1.53)		1.19 (0.86-1.64)	
	Log-additive	---	---	---	1.22 (0.98-1.52)	0.067	1.27 (1.01-1.59)	0.040*

Abbreviations: OR: odds ratio; 95%CI: 95% confidence interval.

* *p* ≤ 0.05 indicates statistical significance.

**Table 5:** Association between *EGFR* tSNPs and risk of astrocytoma under multiple models of inheritance

SNP ID	Model	Genotype	control	case	crude analysis		adjusted by age and gender	
					OR (95% CI)	*p*-value	OR (95% CI)	*p*-value
rs730437	Codominant	A/A	120 (40.3%)	54 (30.9%)	1	0.037	1	0.024*
		C/A	139 (46.6%)	85 (48.6%)	1.36 (0.89-2.07)		1.26 (0.81-1.96)	
		C/C	39 (13.1%)	36 (20.6%)	2.05 (1.18-3.57)		2.27 (1.26-4.10)	
	Dominant	A/A	120 (40.3%)	54 (30.9%)	1	0.039	1	0.072*
		C/A-C/C	178 (59.7%)	121 (69.1%)	1.51 (1.02-2.24)		1.46 (0.96-2.22)	
	Recessive	A/A-C/A	259 (86.9%)	139 (79.4%)	1	0.034	1	0.011*
		C/C	39 (13.1%)	36 (20.6%)	1.72 (1.05-2.83)		2.00 (1.17-3.41)	
	Overdominant	A/A-C/C	159 (53.4%)	90 (51.4%)	1	0.69	1	0.89
		C/A	139 (46.6%)	85 (48.6%)	1.08 (0.74-1.57)		0.97 (0.65-1.45)	
	Log-additive	---	---	---	1.42 (1.08-1.86)	0.011	1.46 (1.10-1.95)	0.0095*
rs11506105	Codominant	A/A	120 (41.1%)	59 (34.1%)	1	0.12	1	0.079*
		A/G	136 (46.6%)	82 (47.4%)	1.23 (0.81-1.86)		1.13 (0.73-1.76)	
		G/G	36 (12.3%)	32 (18.5%)	1.81 (1.02-3.19)		1.99 (1.09-3.63)	
	Dominant	A/A	120 (41.1%)	59 (34.1%)	1	0.13	1	0.21
		A/G-G/G	172 (58.9%)	114 (65.9%)	1.35 (0.91-1.99)		1.30 (0.86-1.96)	
	Recessive	A/A-A/G	256 (87.7%)	141 (81.5%)	1	0.072	1	0.029*
		G/G	36 (12.3%)	32 (18.5%)	1.61 (0.96-2.71)		1.86 (1.07-3.23)	
	Overdominant	A/A-G/G	156 (53.4%)	91 (52.6%)	1	0.86	1	0.74
		A/G	136 (46.6%)	82 (47.4%)	1.03 (0.71-1.51)		0.93 (0.63-1.39)	
	Log-additive	---	---	---	1.32 (1.00-1.73)	0.047	1.34 (1.01-1.80)	0.045*
rs1468727	Codominant	T/T	88 (30.8%)	43 (24.6%)	1	0.041	1	0.022*
		T/C	150 (52.5%)	86 (49.1%)	1.17 (0.75-1.84)		1.08 (0.67-1.74)	
		C/C	48 (16.8%)	46 (26.3%)	1.96 (1.14-3.38)		2.09 (1.17-3.72)	
	Dominant	T/T	88 (30.8%)	43 (24.6%)	1	0.15	1	0.24
		T/C-C/C	198 (69.2%)	132 (75.4%)	1.36 (0.89-2.09)		1.31 (0.84-2.05)	
	Recessive	T/T-T/C	238 (83.2%)	129 (73.7%)	1	0.015	1	0.0062*
		C/C	48 (16.8%)	46 (26.3%)	1.77 (1.12-2.79)		1.99 (1.22-3.24)	
	Overdominant	T/T-C/C	136 (47.5%)	89 (50.9%)	1	0.49	1	0.25
		T/C	150 (52.5%)	86 (49.1%)	0.88 (0.60-1.28)		0.79 (0.53-1.18)	
	Log-additive	---	---	---	1.39 (1.06-1.83)	0.018	1.42 (1.06-1.90)	0.018*

Abbreviations: OR: odds ratio; 95%CI: 95% confidence interval.

* *p* ≤ 0.05 indicates statistical significance.

### Haplotype analysis

**Figure 1:**
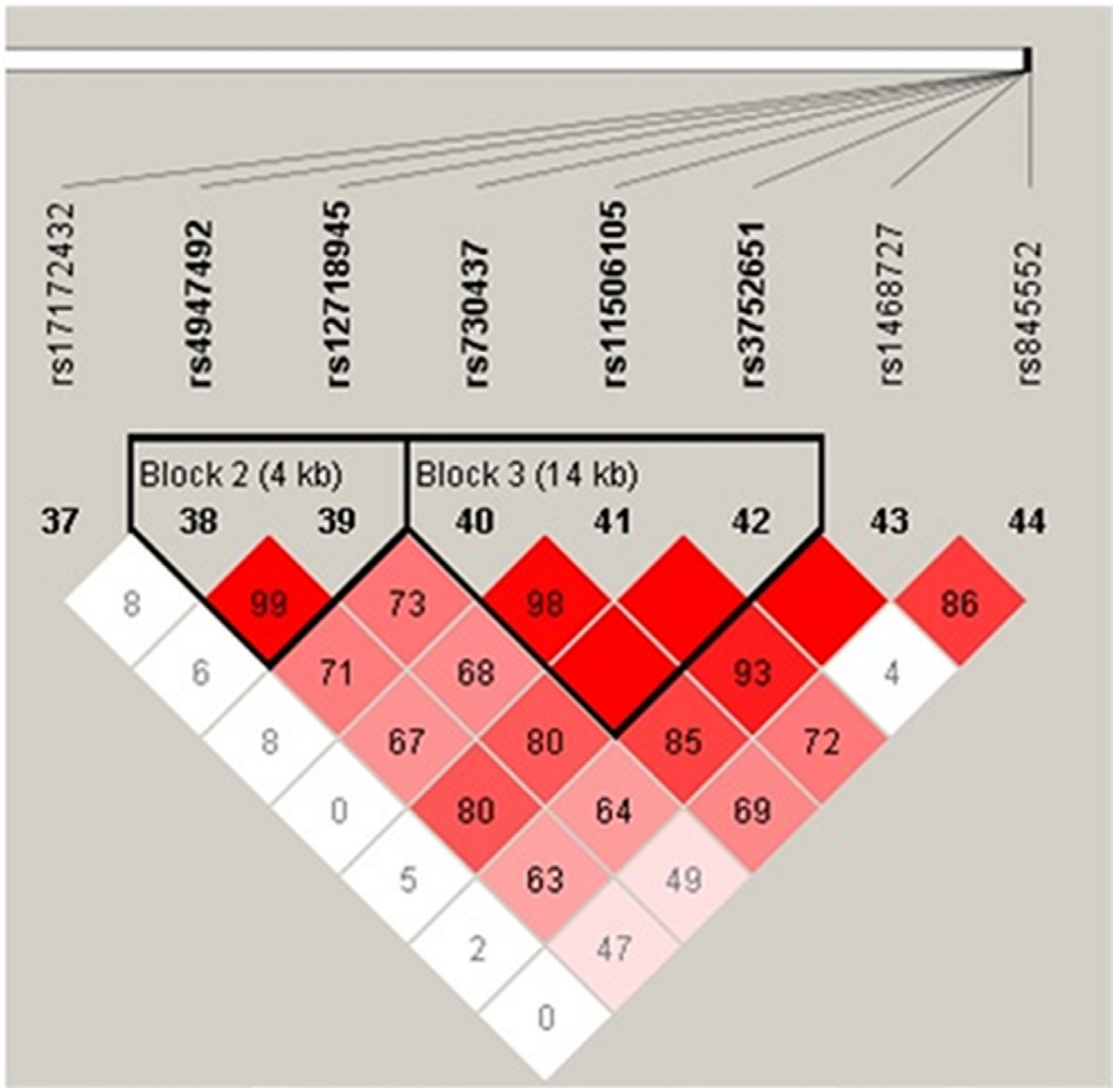
Haplotype block map for tSNPs in the *EGFR* gene

Haplotype analysis detected the existence of two blocks in *EGFR* tSNPs (Figure 1), LD was found between rs4947492 and rs12718945, among rs730437, rs11506105 and rs3752651. Besides, we found that the CGT haplotype was associated with increased glioma risk (OR = 1.41, 95%CI: 1.11-1.79, *p* = 0.006), CGT haplotype and CAT haplotype with an increased risk of astrocytoma (OR = 1.45, 95%CI: 1.07-1.96, *p* = 0.016; OR = 3.11, 95%CI: 1.06-9.10, *p* = 0.039) (Table 6).

**Table 6:** Haplotype frequencies of *EGFR* gene and association with risk of glioma and astrocytoma

rs730437	rs11506105	rs3752651	Glioma			Astrocytoma		
			Freq	OR (95%CI)	*p*-value	Freq	OR (95%CI)	*p*-value
A	A	T	0.5152	1	---	0.5245	1	---
C	G	T	0.3855	1.41	0.006*	0.3781	1.45	0.016*
				(1.11 - 1.79)			(1.07 - 1.96)	
A	A	C	0.0787	1.37	0.16	0.0768	1.34	0.31
				(0.89 - 2.11)			(0.77 - 2.33)	
C	A	T	0.0177	1.84	0.21	0.0173	3.11	0.039*
				(0.72 - 4.73)			(1.06 - 9.10)	

Abbreviations: OR: odds ratio; 95%CI: 95% confidence interval.

* *p* ≤ 0.05 indicates statistical significance.

## DISCUSSION

We successfully genotyped 8 tSNPs in *EGFR* and found some evidence of association at 3 SNPs (rs1468727, rs730437, and rs11506105) that were associated with glioma risk. In addition, CGT and CAT in block 3 trends to increase glioma risk, which suggested that this gene may contribute to glioma in a case-control study from the Han Chinese population. *EGFR* (erbB-1), the prototypical member of the erbB receptor family and is frequently expressed in human tissues. The *EGFR* signaling pathway regulates a wide range of cellular activities associated with cell growth, migration and survival [12]. Several previous studies have assessed single nucleotide polymorphisms (SNPs) in the *EGFR* gene that is implicated in human tumors, such as breast cancer [13], lung cancer [14] and prostate cancer [15]. And several studies have demonstrated that certain genotypes of the *EGFR* gene may be related to glioma susceptibility, which indicated *EGFR* polymorphisms play an important role in the carcinogensis of glioma [11, 16]. Our results found that the C allele of rs1468727 and rs730437, intronic SNPs within the *EGFR* gene, which was associated with an increased risk of glioma compared to controls. Previously, in Chinese populations, Liu et al suggested that the T allele of rs1468727 and C allele of rs730437 were associated with decreased glioma risk [10]; Wang et al observed that the C allele of rs1468727 may increase the risk of glioma, while the A allele of rs730437 may decreased the risk of glioma [11], such inconsistencies in these reports may result from different environment or insufficient sample size. From one study of Andersson U et.al, we found the significant difference exist in these SNPs of our study. For example rs3752651 which significant association with glioma in heterozygote genotype model (OR=1.29, 95%CI = 1.06-1.56, *p*=0.008 ) [16], but in our study it isn't significant (OR=1.13, 95%CI=0.76-1.68, *p*=0.556). A study in Han Chinese population indicated that genotype CC of rs1468727 and rs730437 conferred an elevated risk for glioma [9], which was well in accordance with our findings. All these papers examine implied that two single nucleotide polymorphisms (rs1468727 and rs730437) may play critical roles in the pathogenesis of glioma and might be potential molecular markers for evaluating glioma risk.

The SNP 11506105 in *EGFR* was identified to be associated with an increased risk of glioma, according to allele G association analysis, which is inconsistent with a previous study in a European population, the A allele of rs11506105 had been found to be associated with increased risk of glioma (OR = 1.39, 95%CI: 1.08-1.78, *p* = 0.012) [16]. Our data for rs845552, we did not find any statistical association with astrocytoma risk, suggesting that either there was no such effect or the small sample size after stratified analysis limited statistical power.

Besides the allelic model analysis, we further performed genotypic model analysis to investigate the role of EGFR variants on glioma risk. Our study found that rs730437 and rs1468727 were associated with increased glioma risk in co-dominant, recessive and log-additive models (Table 4). The two SNPs were found to be associated with glioma risk in the many studies. However, the past results were inconsistent. The inconsistency might be attributed to the different ethnicity in the populations studied, given that the minor allele frequency was different. In addition, after a strict Bonferroni correction analysis was applied, the significance level of the association between *EGFR* tSNPs and risk of glioma was attenuated.

There are several inherent limitations that cannot be ignored. Firstly, the subgroup analysis for histology and/or grade, and gender-specific significant variants in glioma patients were not performed, due to the relatively small sample size. Secondly, we selected tSNPs with MAF higher than 5% in the HapMap Chinese Han Beijing population to determine the statistical power was large enough for analyzing data, the haplotype-based study was also performed to affirm sufficiently high power to detect the association between candidate tSNPs and glioma risk. Thirdly, glioma patients and controls were drawn from same hospital, so selection bias cannot be excluded and the subjects might not be representative of the general population.

## CONCLUSIONS

Our study is an exploratory research which shed new light on the association between *EGFR* tSNPs and glioma risk in the Chinese population. Combined with the previous studies, we only further ascertain the potential role of the *EGFR* gene to glioma onset. Further investigation with a larger sample size in Chinese populations or another ethnic is warranted.

## MATERIALS AND METHODS

### Study population

We recruited patients between December 2002 and April 2013 for the molecular epidemiology study at the First Affiliated Hospital of the Medical College of Xi’an Jiaotong University. All gliomas cases were confirmed by the newly diagnosed histopathologically, and patients who had any history of cancer and received either radiotherapy or chemotherapy before surgery were excluded. Through our normal the epidemiological investigation and eliminated individuals with any glioma disease family history of more than three generations, eventually a total of 298 controls during the similar period was randomly recruited from the medical examination center at First Affiliated Hospital. Their frequency matched to all adult brain tumor cases on age, sex, and region. These controls were cancer-free and genetically unrelated to the patients.

### Data collection

Each participant was interviewed by a trained nurse, a standard questionnaire was used to collect detailed information regarding demographic characteristics, radiation exposure, smoking habit, alcohol drinking habit, family history of cancer and other factors. Each subject donated 3-5 ml of venous blood, and all subjects who agreed to the purpose and experimental procedures of the study, and signed their written informed consents prior to sample donation. The research protocol was performed in accordance with the Declaration of Helsinki and approved by the Human Research Committee of the First Affiliated Hospital of the Medical College of Xi’an Jiaotong University for Approval of Research Involving Human Subjects.

### SNP selection and genotyping

Using the public dbSNP (https://www.ncbi.nlm.nih.gov/snp) and HapMap (http://www.hapmap.org/) and identify linkage disequilibrium (LD) blocks within *EGFR* to get a dense tagging of SNPs, we used Haploview software (http://www.broad.mit.edu/mpg/haploview/) setting the minimum r2 to 0.9 and the minimum minor allele frequency to 5 % in HapMap Chinese Han Beijing residents. Finally we selected candidate 8 tSNPs in the *EGFR* gene for genotyping, which previously reported being associated with gliomas [16]. Genomic DNA was extracted from whole blood using the GoldMag® nanoparticles method (GoldMag Ltd. Xi’an, China) according to the manufacturer's instructions, and the DNA concentration was measured with the NanoDrop 2000C (Thermo Scientific, Waltham, Massachusetts, USA). The Sequenom MassARRAY Assay Design 3.0 Software (San Diego, California, USA) was used to design Multiplexed SNP MassEXTEND assays [17]. SNPs genotyping was performed by the Sequenom MassARRAY RS1000 (San Diego, California, USA) and Sequenom Typer 4.0 Software (San Diego, California, USA) was used to perform data management and analysis as previously described [17, 18].

### Data analysis

Statistical calculations were made using Microsoft Excel (Redmond, WA, USA) and SPSS 19.0 statistical package (SPSS, Chicago, IL, USA). All statistical tests were two-sided, and *p* ≤ 0.05 was regarded as statistically significant differences. Each tSNP frequency was assessed for departure from Hardy–Weinberg Equilibrium (HWE) using an exact test among controls. We compared genotype frequencies and allele frequencies between cases and controls using the χ^2^ test. To estimate the association between the *EGFR* genetic polymorphisms and risk of glioma, as measured by odds ratios (ORs) and 95% confidence intervals (CIs) were calculated using unconditional logistic regression with adjustment for age and gender [19]. We used multiple inheritance models (codominant, dominant, recessive, overdominant and additive) to evaluate the associations between certain tSNPs in *EGFR* gene and glioma risk, with SNPstats software (http://bioinfo.iconcologia.net/snpstats/start.htm) [20]. For each polymorphism, ORs and 95% CIs were calculated by unconditional logistic regression analysis with and without adjustment of age and gender [19].

Finally, we used Haploview software package (version 4.2) to evaluate the pairwise linkage disequilibrium (LD), haplotype construction, and genetic association at polymorphism loci [21, 22].
